# Pyridinium tosyl­ate

**DOI:** 10.1107/S2414314624008319

**Published:** 2024-08-30

**Authors:** Eric Cyriel Hosten, Richard Betz

**Affiliations:** a Nelson Mandela University, Summerstrand Campus, Department of Chemistry, University Way, Summerstrand, PO Box 77000, Port Elizabeth, 6031, South Africa; University of Aberdeen, United Kingdom

**Keywords:** crystal structure, hydrogen bond, pyridinium salt

## Abstract

The title compound (systematic name: pyridinium 4-methyl­benzene­sulfonate), C_5_H_6_N^+^·C_7_H_7_O_3_S^−^, is the pyridinium salt of *para*-toluene­sulfonic acid. In the crystal, classical N—H⋯O hydrogen bonds as well as C—H⋯O contacts connect the cationic and anionic entities into sheets lying parallel to the *ab* plane.

## Abstract 2

In the crystal, classical N—H⋯O hydrogen bonds as well as C—H⋯O contacts connect the cationic and anionic entities into sheets lying parallel to the *ab* plane.

## Graphical abstract

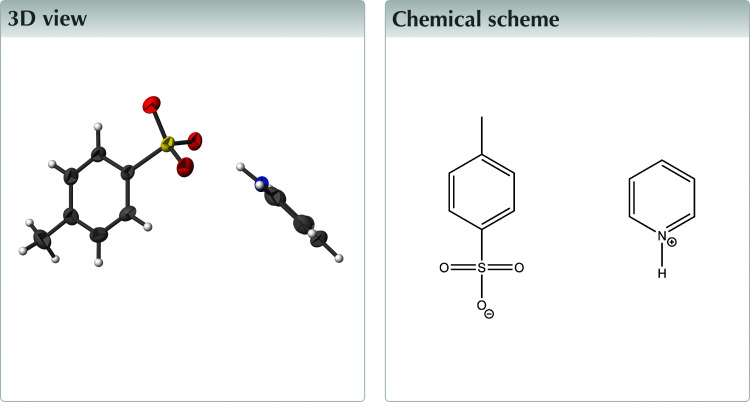

## Structure description

Many fundamental synthesis reactions in preparative organic chemistry make use of activated reagents to allow for the faster and easier production of certain key compounds or to avoid the presence of cumbersome equilibrium reactions. A prime example for this finding is a series of derivatives of carb­oxy­lic acids such as esters and amides that – instead of employing the free acid as staring material – are often more conveniently obtained by using the pertaining carb­oxy­lic anhydride or acyl chloride or bromide as starting materials (Becker *et al.*, 2000 ▸). One downside of this increased reactivity is the frequent need to use auxiliary reagents that can mitigate potential side effects of the byproducts produced, most notably basic reagents that can act as acid scavengers to prevent undesired hydrolysis effects. Among the more common ingredients used in the latter context are amines such as tri­ethyl­amine or pyridine whose onium salts can often conveniently be removed from reaction mixtures in organic solvents by means of simple filtration. Occasionally, however, some of the material tenaciously migrates through many steps of purification procedures and can manifest as lingering impurity in the assumed final product. To prevent the waste of valuable data-collection time on diffractometers for future researchers, it is of importance to report the structures even of such undesired compounds as a reference point for the broader scientific community, as done previously by us for ammonium formate (Hosten & Betz, 2014 ▸), ammonium phenyl glyoxylate (Hosten & Betz, 2015 ▸) as well as the chlorides (Maritz *et al.*, 2021 ▸; Muller *et al.*, 2021*a* ▸,*b* ▸,*c* ▸) and tosyl­ate salts (Moleko *et al.*, 2015 ▸) of a number of protonated amines. Furthermore, the mol­ecular and crystal structures of the non-radioactive halogenide salts of the pyridinium cation are apparent in the literature (Boenigk & Mootz, 1988 ▸; Mootz & Hocken, 1989 ▸; Klooster *et al.*, 2019 ▸; Owczarek *et al.*, 2012 ▸).

**Figure 1:**
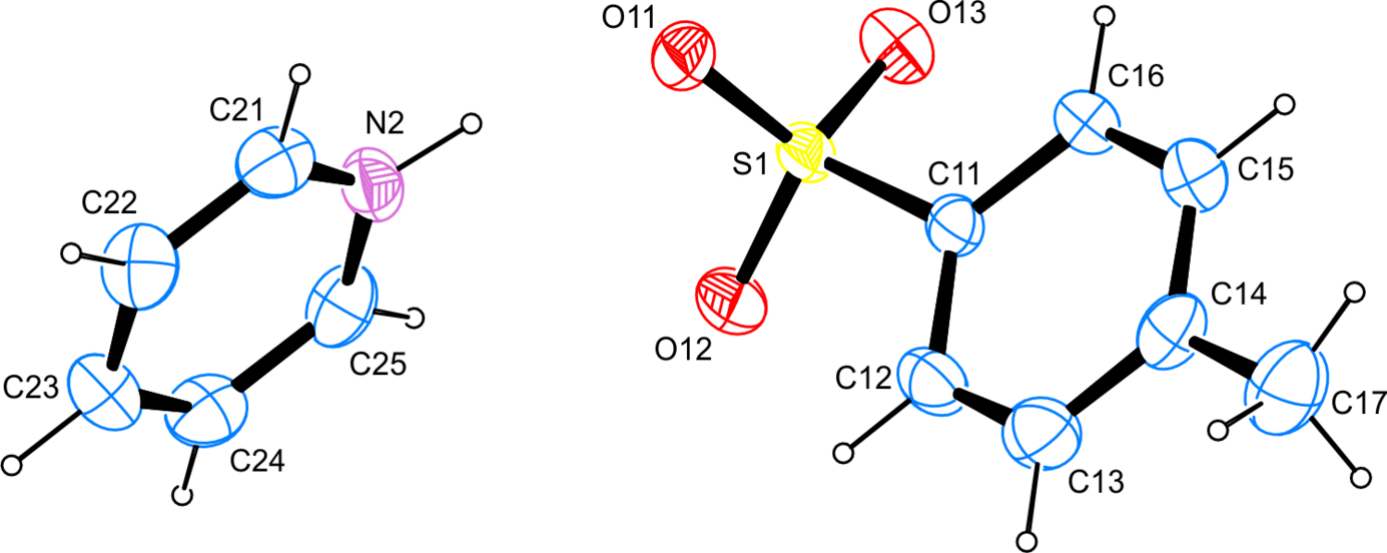
The mol­ecular structure of the title compound, with anisotropic displacement ellipsoids drawn at 50% probability level.

The asymmetric unit of the title compound, C_5_H_6_N^+^·C_7_H_7_O_3_S^−^, is shown in Fig. 1 ▸ and consists of one complete ion pair. The S—O bond lengths in the anion are found in the narrow range of 1.4525 (14)–1.4682 (14) Å, which is in agreement with full resonant delocalization of the anionic charge over all three oxygen atoms. All other bond lengths and angles are found in good agreement with other tosyl­ates whose mol­ecular and crystal structures were determined on grounds of diffraction studies conducted on single crystals and whose metrical parameters have been deposited with the Cambridge Structural Database (Allen, 2002 ▸). The least-squares planes as defined by the non-hydrogen atoms of the cation as well as the intra­cyclic carbon atoms of the tosyl­ate anion inter­sect at an angle of 74.44 (10)°, *i.e.* the two separate aromatic systems in the asymmetric unit are orientated almost perpendicular to one another.

**Figure 2:**
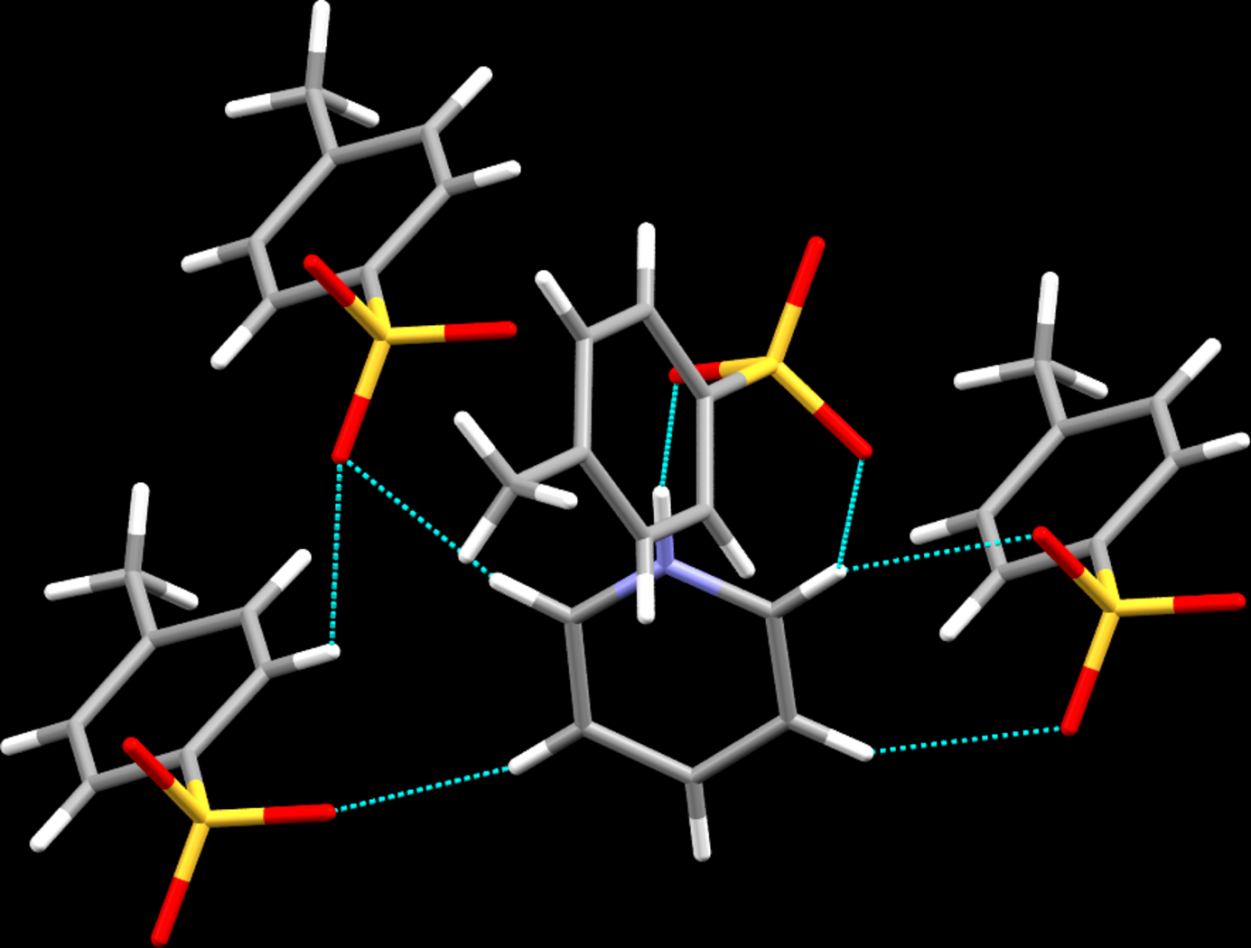
Inter­molecular contacts, viewed approximately along [001].

In the crystal, classical N—H⋯O hydrogen bonds are observed as well as C—H⋯O contacts whose range falls by more than 0.1 Å below the sum of van der Waals radii of the atoms participating in them (Table 1 ▸). While the classical hydrogen bonds are established by the pnictogen-bonded hydrogen atom as donor and one of the oxygen atoms of the sulfato group as acceptor, the C—H⋯O contacts are supported by each of the aromatic hydrogen atoms of the cation except for the one in *para* position to the protonated nitro­gen atom. All three sulfur-bonded oxygen atoms act as acceptors in for the latter contacts. In terms of graph-set analysis (Etter *et al.*, 1990 ▸; Bernstein *et al.*, 1995 ▸), the descriptor for the classical hydrogen bonds is *D* on the unary level while the C—H⋯O contacts require a *DDDD* descriptor on the same level. Overall, the inter­molecular contacts connect the ions of the title compound into sheets lying parallel the the *ab* plane. A depiction of the pattern is shown in Fig. 2 ▸. Aromatic π–π stacking is not a prominent feature in the crystal structure of the title compound with the shortest inter­centroid distance between two aromatic systems measuring 4.9276 (12) Å for the anion and its symmetry-generated equivalent.

## Synthesis and crystallization

After an initial unintentional isolation of the crystalline compound from a different synthesis product the compound was targeted by reacting a slight excess of liquid pyridine with solid tosylic acid in solvent-free conditions. Crystals of the title compound in the form of colourless blocks suitable for the diffraction study were obtained upon free evaporation of the reaction mixture at room temperature.

## Refinement

Data collection and crystallographic data are summarized in Table 2 ▸. The crystal used for data collection was found to be an an inversion twin with a volume ratio of 79.3:20.7.

## Supplementary Material

Crystal structure: contains datablock(s) I, global. DOI: 10.1107/S2414314624008319/hb4482sup1.cif

Structure factors: contains datablock(s) I. DOI: 10.1107/S2414314624008319/hb4482Isup2.hkl

Supporting information file. DOI: 10.1107/S2414314624008319/hb4482Isup3.cml

CCDC reference: 2379208

Additional supporting information:  crystallographic information; 3D view; checkCIF report

## Figures and Tables

**Table 1:** Hydrogen-bond geometry (Å, °)

*D*—H⋯*A*	*D*—H	H⋯*A*	*D*⋯*A*	*D*—H⋯*A*
N2—H2⋯O11	0.93 (3)	1.81 (3)	2.724 (2)	166 (3)
C21—H21⋯O13^i^	0.95	2.41	3.306 (2)	157
C22—H22⋯O12^ii^	0.95	2.36	3.117 (2)	136
C24—H24⋯O13^iii^	0.95	2.35	3.202 (2)	149
C25—H25⋯O12	0.95	2.54	3.259 (3)	133
C25—H25⋯O11^iii^	0.95	2.36	3.194 (2)	147

Symmetry codes: (i) 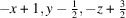; (ii) 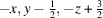; (iii) 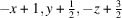.

**Table 2:** Experimental details

Crystal data	
Chemical formula	C_5_H_6_N^+^·C_7_H_7_O_3_S^−^
*M*_r_	251.29
Crystal system, space group	Orthorhombic, *P*2_1_2_1_2_1_
Temperature (K)	200
*a*, *b*, *c* (Å)	5.8868 (2), 8.8927 (4), 22.8226 (9)
*V* (Å^3^)	1194.75 (8)
*Z*	4
Radiation type	Mo *K*α
μ (mm^−1^)	0.27
Crystal size (mm)	0.57 × 0.39 × 0.34

Data collection	
Diffractometer	Bruker APEXII CCD
Absorption correction	Numerical (*SADABS*; Krause *et al.*, 2015 ▸)
*T*_min_, *T*_max_	0.904, 1.000
No. of measured, independent and observed [*I* > 2σ(*I*)] reflections	11137, 2972, 2903
*R*_int_	0.013
(sin θ/λ)_max_ (Å^−1^)	0.668

Refinement	
*R*[*F*^2^ > 2σ(*F*^2^)], *wR*(*F*^2^), *S*	0.027, 0.072, 1.08
No. of reflections	2972
No. of parameters	160
H-atom treatment	H atoms treated by a mixture of independent and constrained refinement
Δρ_max_, Δρ_min_ (e Å^−3^)	0.27, −0.26
Absolute structure	Refined as an inversion twin.
Absolute structure parameter	0.21 (8)

Computer programs: *APEX2* and *SAINT* (Bruker, 2010 ▸), *SHELXS97*, *SHELXL97* and *SHELXTL* (Sheldrick, 2008 ▸).

## full crystallographic data

### Pyridinium 4-methylbenzenesulfonate Crystal data

**Table:**

C_5_H_6_N^+^·C_7_H_7_O_3_S^−^	*D*_x_ = 1.397 Mg m^−^^3^
*M**_r_* = 251.29	Mo *K*α radiation, λ = 0.71073 Å
Orthorhombic, *P*2_1_2_1_2_1_	Cell parameters from 9608 reflections
*a* = 5.8868 (2) Å	θ = 2.5–28.3°
*b* = 8.8927 (4) Å	µ = 0.27 mm^−^^1^
*c* = 22.8226 (9) Å	*T* = 200 K
*V* = 1194.75 (8) Å^3^	Block, colourless
*Z* = 4	0.57 × 0.39 × 0.34 mm
*F*(000) = 528	

### Pyridinium 4-methylbenzenesulfonate Data collection

**Table:**

Bruker APEXII CCD diffractometer	2972 independent reflections
Radiation source: sealed tube	2903 reflections with *I* > 2σ(*I*)
Graphite monochromator	*R*_int_ = 0.013
φ and ω scans	θ_max_ = 28.3°, θ_min_ = 2.5°
Absorption correction: numerical (SADABS; Krause *et al.*, 2015)	*h* = −7→7
*T*_min_ = 0.904, *T*_max_ = 1.000	*k* = −11→11
11137 measured reflections	*l* = −30→30

### Pyridinium 4-methylbenzenesulfonate Refinement

**Table:**

Refinement on *F*^2^	Secondary atom site location: difference Fourier map
Least-squares matrix: full	Hydrogen site location: mixed
*R*[*F*^2^ > 2σ(*F*^2^)] = 0.027	H atoms treated by a mixture of independent and constrained refinement
*wR*(*F*^2^) = 0.072	*w* = 1/[σ^2^(*F*_o_^2^) + (0.0366*P*)^2^ + 0.2871*P*] where *P* = (*F*_o_^2^ + 2*F*_c_^2^)/3
*S* = 1.08	(Δ/σ)_max_ = 0.001
2972 reflections	Δρ_max_ = 0.27 e Å^−^^3^
160 parameters	Δρ_min_ = −0.26 e Å^−^^3^
0 restraints	Absolute structure: Refined as an inversion twin.
Primary atom site location: structure-invariant direct methods	Absolute structure parameter: 0.21 (8)

### Pyridinium 4-methylbenzenesulfonate Special details

**Table:**

Geometry. All esds (except the esd in the dihedral angle between two l.s. planes) are estimated using the full covariance matrix. The cell esds are taken into account individually in the estimation of esds in distances, angles and torsion angles; correlations between esds in cell parameters are only used when they are defined by crystal symmetry. An approximate (isotropic) treatment of cell esds is used for estimating esds involving l.s. planes.
Refinement. Refined as a 2-component inversion twin. The N-bonded H atom was located in a difference map and refined freely. The aromatic carbon-bound H atoms were placed in calculated positions (C—H = 0.95 Å) and were included in the refinement in the riding model approximation, with *U*(H) set to 1.2*U*_eq_(C). The H atoms of the methyl group were allowed to rotate but not to tip around the C—C bond to best fit the experimental electron density with *U*(H) set to 1.5*U*_eq_(C).

### Pyridinium 4-methylbenzenesulfonate Fractional atomic coordinates and isotropic or equivalent isotropic displacement parameters (Å^2^)

**Table:**

	*x*	*y*	*z*	*U*_iso_*/*U*_eq_	
S1	0.59831 (7)	0.68244 (5)	0.67195 (2)	0.02552 (11)	
O11	0.5179 (3)	0.56756 (15)	0.71323 (6)	0.0338 (3)	
O12	0.5035 (3)	0.82991 (15)	0.68422 (6)	0.0367 (3)	
O13	0.8442 (2)	0.68221 (16)	0.66646 (6)	0.0345 (3)	
N2	0.1743 (3)	0.6291 (2)	0.78954 (7)	0.0330 (4)	
C11	0.4869 (3)	0.62497 (19)	0.60326 (8)	0.0257 (3)	
C12	0.2780 (3)	0.6787 (2)	0.58477 (9)	0.0335 (4)	
H12	0.200255	0.752191	0.607339	0.040*	
C13	0.1821 (4)	0.6250 (3)	0.53319 (10)	0.0390 (5)	
H13	0.039834	0.663586	0.520560	0.047*	
C14	0.2902 (4)	0.5162 (2)	0.49988 (9)	0.0357 (4)	
C15	0.5018 (4)	0.4649 (2)	0.51834 (9)	0.0369 (4)	
H15	0.580051	0.392004	0.495557	0.044*	
C16	0.6006 (4)	0.5186 (2)	0.56962 (8)	0.0331 (4)	
H16	0.745310	0.482637	0.581603	0.040*	
C17	0.1802 (5)	0.4560 (3)	0.44461 (10)	0.0515 (6)	
H17A	0.185227	0.533185	0.413990	0.077*	
H17B	0.021716	0.429478	0.452787	0.077*	
H17C	0.262372	0.366456	0.431323	0.077*	
C21	0.0159 (4)	0.5375 (2)	0.81078 (9)	0.0345 (4)	
H21	0.032091	0.431632	0.806892	0.041*	
C22	−0.1711 (4)	0.5966 (2)	0.83835 (9)	0.0375 (5)	
H22	−0.286699	0.532290	0.853071	0.045*	
C23	−0.1893 (4)	0.7505 (3)	0.84444 (10)	0.0374 (5)	
H23	−0.317615	0.793002	0.863489	0.045*	
C24	−0.0202 (4)	0.8426 (2)	0.82274 (9)	0.0362 (4)	
H24	−0.029529	0.948643	0.827267	0.043*	
C25	0.1609 (4)	0.7788 (2)	0.79463 (9)	0.0351 (5)	
H25	0.277146	0.840648	0.778725	0.042*	
H2	0.297 (5)	0.594 (3)	0.7673 (12)	0.054 (8)*	

### Pyridinium 4-methylbenzenesulfonate Atomic displacement parameters (Å^2^)

**Table:**

	*U*^11^	*U*^22^	*U*^33^	*U*^12^	*U*^13^	*U*^23^
S1	0.0293 (2)	0.02035 (18)	0.02696 (19)	0.00397 (16)	0.00644 (17)	0.00092 (16)
O11	0.0426 (8)	0.0287 (7)	0.0301 (6)	0.0043 (6)	0.0093 (6)	0.0064 (5)
O12	0.0488 (8)	0.0249 (6)	0.0364 (7)	0.0115 (6)	0.0066 (6)	−0.0023 (6)
O13	0.0295 (6)	0.0324 (6)	0.0417 (7)	0.0001 (5)	0.0046 (5)	−0.0054 (6)
N2	0.0307 (8)	0.0418 (9)	0.0265 (7)	0.0042 (7)	0.0012 (6)	−0.0062 (7)
C11	0.0274 (8)	0.0227 (7)	0.0270 (8)	0.0012 (6)	0.0070 (7)	0.0037 (6)
C12	0.0261 (8)	0.0338 (9)	0.0407 (10)	0.0044 (8)	0.0068 (7)	0.0008 (9)
C13	0.0289 (9)	0.0449 (12)	0.0433 (11)	0.0019 (8)	0.0006 (9)	0.0057 (9)
C14	0.0388 (11)	0.0389 (11)	0.0294 (9)	−0.0051 (9)	0.0009 (8)	0.0073 (8)
C15	0.0423 (11)	0.0369 (10)	0.0316 (9)	0.0076 (9)	0.0051 (8)	−0.0028 (8)
C16	0.0341 (9)	0.0337 (9)	0.0316 (8)	0.0104 (8)	0.0041 (8)	−0.0001 (7)
C17	0.0554 (15)	0.0636 (16)	0.0356 (11)	−0.0051 (13)	−0.0077 (10)	0.0004 (11)
C21	0.0447 (11)	0.0244 (9)	0.0345 (9)	−0.0013 (8)	−0.0069 (8)	−0.0036 (7)
C22	0.0370 (10)	0.0334 (10)	0.0423 (11)	−0.0134 (8)	0.0044 (9)	0.0015 (8)
C23	0.0315 (10)	0.0385 (11)	0.0422 (11)	0.0040 (8)	0.0039 (9)	−0.0069 (9)
C24	0.0480 (11)	0.0216 (8)	0.0390 (10)	−0.0030 (7)	−0.0055 (9)	−0.0015 (8)
C25	0.0370 (11)	0.0377 (10)	0.0305 (9)	−0.0133 (8)	0.0000 (8)	0.0050 (7)

### Pyridinium 4-methylbenzenesulfonate Geometric parameters (Å, º)

**Table:**

S1—O12	1.4525 (14)	C15—C16	1.391 (3)
S1—O13	1.4527 (14)	C15—H15	0.9500
S1—O11	1.4682 (14)	C16—H16	0.9500
S1—C11	1.7745 (19)	C17—H17A	0.9800
N2—C21	1.330 (3)	C17—H17B	0.9800
N2—C25	1.339 (3)	C17—H17C	0.9800
N2—H2	0.93 (3)	C21—C22	1.373 (3)
C11—C12	1.385 (3)	C21—H21	0.9500
C11—C16	1.390 (2)	C22—C23	1.380 (3)
C12—C13	1.390 (3)	C22—H22	0.9500
C12—H12	0.9500	C23—C24	1.381 (3)
C13—C14	1.385 (3)	C23—H23	0.9500
C13—H13	0.9500	C24—C25	1.368 (3)
C14—C15	1.392 (3)	C24—H24	0.9500
C14—C17	1.516 (3)	C25—H25	0.9500
			
O12—S1—O13	113.63 (9)	C11—C16—C15	119.79 (19)
O12—S1—O11	112.36 (9)	C11—C16—H16	120.1
O13—S1—O11	112.06 (9)	C15—C16—H16	120.1
O12—S1—C11	106.76 (9)	C14—C17—H17A	109.5
O13—S1—C11	106.96 (8)	C14—C17—H17B	109.5
O11—S1—C11	104.32 (9)	H17A—C17—H17B	109.5
C21—N2—C25	122.43 (19)	C14—C17—H17C	109.5
C21—N2—H2	122.5 (18)	H17A—C17—H17C	109.5
C25—N2—H2	114.9 (18)	H17B—C17—H17C	109.5
C12—C11—C16	119.60 (18)	N2—C21—C22	119.67 (18)
C12—C11—S1	119.87 (14)	N2—C21—H21	120.2
C16—C11—S1	120.39 (15)	C22—C21—H21	120.2
C11—C12—C13	120.01 (19)	C21—C22—C23	119.2 (2)
C11—C12—H12	120.0	C21—C22—H22	120.4
C13—C12—H12	120.0	C23—C22—H22	120.4
C14—C13—C12	121.2 (2)	C22—C23—C24	119.8 (2)
C14—C13—H13	119.4	C22—C23—H23	120.1
C12—C13—H13	119.4	C24—C23—H23	120.1
C13—C14—C15	118.3 (2)	C25—C24—C23	118.95 (18)
C13—C14—C17	120.5 (2)	C25—C24—H24	120.5
C15—C14—C17	121.3 (2)	C23—C24—H24	120.5
C16—C15—C14	121.1 (2)	N2—C25—C24	119.96 (19)
C16—C15—H15	119.5	N2—C25—H25	120.0
C14—C15—H15	119.5	C24—C25—H25	120.0
			
O12—S1—C11—C12	−27.38 (17)	C13—C14—C15—C16	1.5 (3)
O13—S1—C11—C12	−149.35 (15)	C17—C14—C15—C16	−178.9 (2)
O11—S1—C11—C12	91.76 (16)	C12—C11—C16—C15	−1.2 (3)
O12—S1—C11—C16	156.98 (15)	S1—C11—C16—C15	174.43 (16)
O13—S1—C11—C16	35.00 (18)	C14—C15—C16—C11	0.1 (3)
O11—S1—C11—C16	−83.89 (16)	C25—N2—C21—C22	1.0 (3)
C16—C11—C12—C13	0.7 (3)	N2—C21—C22—C23	−1.1 (3)
S1—C11—C12—C13	−174.98 (16)	C21—C22—C23—C24	0.1 (4)
C11—C12—C13—C14	1.0 (3)	C22—C23—C24—C25	1.1 (3)
C12—C13—C14—C15	−2.0 (3)	C21—N2—C25—C24	0.3 (3)
C12—C13—C14—C17	178.4 (2)	C23—C24—C25—N2	−1.3 (3)

### Pyridinium 4-methylbenzenesulfonate Hydrogen-bond geometry (Å, º)

**Table:**

*D*—H···*A*	*D*—H	H···*A*	*D*···*A*	*D*—H···*A*
N2—H2···O11	0.93 (3)	1.81 (3)	2.724 (2)	166 (3)
C21—H21···O13^i^	0.95	2.41	3.306 (2)	157
C22—H22···O12^ii^	0.95	2.36	3.117 (2)	136
C24—H24···O13^iii^	0.95	2.35	3.202 (2)	149
C25—H25···O12	0.95	2.54	3.259 (3)	133
C25—H25···O11^iii^	0.95	2.36	3.194 (2)	147

Symmetry codes: (i) −*x*+1, *y*−1/2, −*z*+3/2; (ii) −*x*, *y*−1/2, −*z*+3/2; (iii) −*x*+1, *y*+1/2, −*z*+3/2.
